# Formation of Multi-Component Extracellular Matrix Protein Fibers

**DOI:** 10.1038/s41598-018-20371-8

**Published:** 2018-01-30

**Authors:** Seungkuk Ahn, Keel Yong Lee, Kevin Kit Parker, Kwanwoo Shin

**Affiliations:** 1 Department of Chemistry and Institute of Biological Interfaces, Sogang University, Seoul, 121-742 Republic of Korea; 2 Disease Biophysics Group, Wyss Institute for Biologically Inspired Engineering, John A. Paulson School of Engineering and Applied Sciences, Harvard University, 29 Oxford St., Pierce Hall 321, Cambridge, MA 02138 USA

## Abstract

The extracellular matrix (ECM) consists of polymerized protein monomers that form a unique fibrous network providing stability and structural support to surrounding cells. We harnessed the fibrillogenesis mechanisms of naturally occurring ECM proteins to produce artificial fibers with a heterogeneous protein makeup. Using ECM proteins as fibril building blocks, we created uniquely structured multi-component ECM fibers. Sequential incubation of fibronectin (FN) and laminin (LAM) resulted in self-assembly into locally stacked fibers. In contrast, simultaneous incubation of FN with LAM or collagen (COL) produced molecularly stacked multi-component fibers because both proteins share a similar assembly mechanism or possess binding domains specific to each other. Sequential incubation of COL on FN fibers resulted in fibers with sandwiched layers because COL molecules bind to the external surface of FN fibers. By choosing proteins for incubation according to the interplay of their fibrillogenesis mechanisms and their binding domains (exposed when they unfold), we were able to create ECM protein fibers that have never before been observed.

## Introduction

The animal extracellular matrix (ECM) is a heterogeneous connective fiber network composed of various fibrous glycoproteins, proteoglycans (PGs), and small molecules^1–5^. In the ECM’s anionic environment (created by the presence of polyanionic PGs containing carboxyl and sulfonyl groups), glycoproteins near the basal region of the cellular membrane, such as fibronectin (FN), collagen (COL), laminin (LAM), and elastin (ELAS), are polymerized into fibers to form ECM networks that coordinate *in vivo* to provide the physical scaffolding, mechanical stability, and intercellular communication necessary for tissue morphogenesis and homeostasis^1–8^.

Fibrillogenesis occurs in a tissue-specific manner and at different times. For example, fibrillogenensis of FN and LAM, which are large ECM glycoproteins rich in β-sheet structures, is initiated when they bind to specific binding domains in integrin or in negatively charged compounds such as heparan sulfate^7–15^. Once FN or LAM is bound to the receptor, the surrounding FN and LAM proteins undergo a conformational change from a globular to an unfolded state, resulting in fiber extension^7–15^. Fibrillogenesis of FN or LAM is thus a two-step process: self-assembly (propagation step) followed by glycoprotein unfolding (initiation step). The process fundamentally resembles the addition polymerization process often observed in synthetic polymerization. In contrast, ECM proteins that lack β-sheet structures, such as COL and ELAS, are assembled into networks via cross-linking, a process that requires cross-linking agents or specific conditions such as appropriate pH and temperature^7,16–19^.

Several attempts have been made to explain what drives fibrillogenesis of FN or LAM. For example, external mechanical forces or *in vitro* electrostatic surface charges have been proposed^20–27^. Essentially, the process begins with conformational unfolding of the glycoproteins, followed by spontaneous fibrillogenesis to form FM or LAM fibers. This process depends on the presence of an anionic environment, which is provided by PGs in the ECM.

Currently, only fibrillogenesis of single ECM protein fibers has been investigated^20–27^. In nature, though, the ECM architecture is built from multiple ECM proteins and exhibits complex dynamics. The ability to create custom-engineered ECM microenvironments with chosen ECM proteins would be a great help in studying the structural and biochemical properties governing fiber-fiber and fiber-cell interplay. Therefore, we investigated the formation of multi-component ECM protein fibers, and specifically whether fibrillogenensis mechanisms and protein binding sites could be chosen to form a specific type of multi-component fiber.

**Figure 1:**
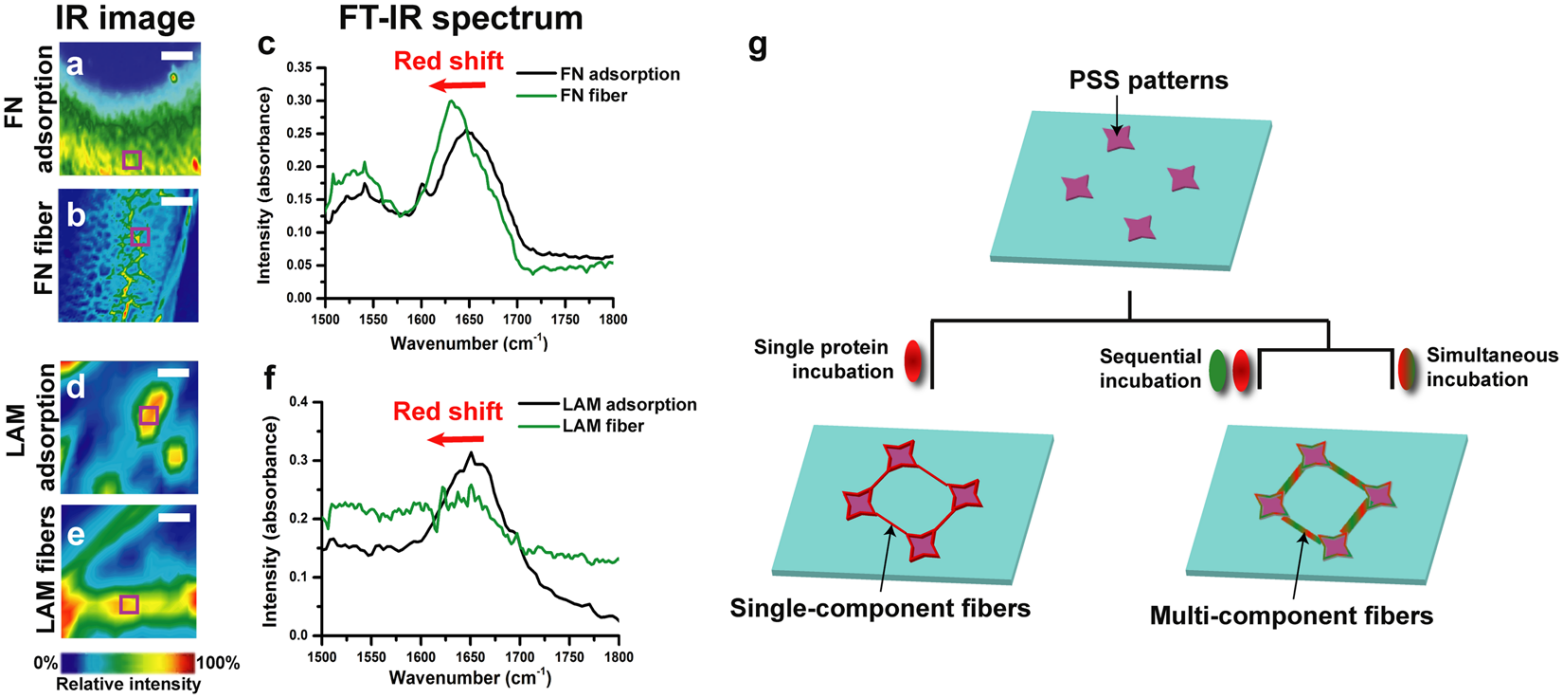
Analysis of charge-induced self-assembly of single-component FN and LAM fibers and overview of incubation process. (**a**–**f**) FT-IR analysis of amide I peak shifts from IR images of adsorbed ECM proteins and ECM fibers obtained following single incubation of proteins on a spin-coated PSS surface: IR images for (**a**) FN adsorption and (**b**) FN fibers and (**c**) FTIR spectra, where the scales of (**a**) and (**b**) are 80 and 160 µm, respectively. IR images for (**d**) LAM adsorption, (**e**) LAM fibers, and FT-IR spectra, where the scales of (**d**) and (**e**) are both 20 µm. (**g**) Schematic diagram of the incubation processes used to produce single-component and multi-component protein fibers.

We hypothesized that if fibrillogenesis of two ECM proteins could be initiated and propagated on a charged surface in a similar manner, then the proteins would assemble into a single fiber. For our model components we chose FN, LAM, COL, and ELAS. Fibers were formed by incubating these proteins singly, sequentially, or simultaneously on two types of surfaces, one spin-coated with polystyrene sulfonated acid (PSS) and the other printed with PSS micro-patterns (Fig. 1g; see Methods section). Negatively charged synthetic polymer or phospholipid surfaces have been previously used to replicate the negative surface charge of PGs, enabling the extension and polymerization of FN or LAM molecules into large fibrillar networks^22–30^. Recently, we observed large-scale self-organizing single ECM protein networks on PG-mimicking PSS^28^. PSS works as a mimetic of PG because it possesses a functional group in common with PG: a sulfonyl group.^29,30^ The sequence of negatively charged groups in PSS attracts the positively charged domains in ECM proteins and induces protein unfolding, leading to fibrillogenesis. Only β-sheet–rich ECM proteins (FN and LAM) formed a long-ranged fibrillar structure; β-sheet–poor ECM proteins (COL and ELAS) did not.

## Results

We discovered that the negatively charged PSS surface induced protein unfolding of FN and LAM and subsequent self-assembly into fiber networks but did not have the same effect on COL fibers or adsorbed ELAS fibers, which instead were randomly distributed (see Supplementary Fig. S1a–f). These inferences were verified by comparing the amide I peaks (which are highly sensitive to changes in protein secondary structures^31^) for the IR images of native FN and LAM and fibers induced on a spin-coated PSS surface (Fig. 1a–f). The amide I peaks of FN and LAM fibers were red-shifted (Fig. 1c,f), indicating that both proteins unfolded upon binding to a charged domain^28,32,33^, but the amide I peaks of the randomly distributed COL fibers and ELAS adsorbates revealed no red shift (see Supplementary Fig. S1c,f). These different fibrillogenesis characteristics enable diverse *in vivo* combinations of composite ECM protein networks. FN and LAM were also found to form controlled networks among PSS islands (see Supplementary Fig. S1,g,h), while COL and ELAS formed random fibers unrelated to the islands (see Supplementary Fig. S1,i,j). These structures reflect the natural tendencies of different ECM proteins to form fibers based on the presence or absence of a surface charge.

**Figure 2:**
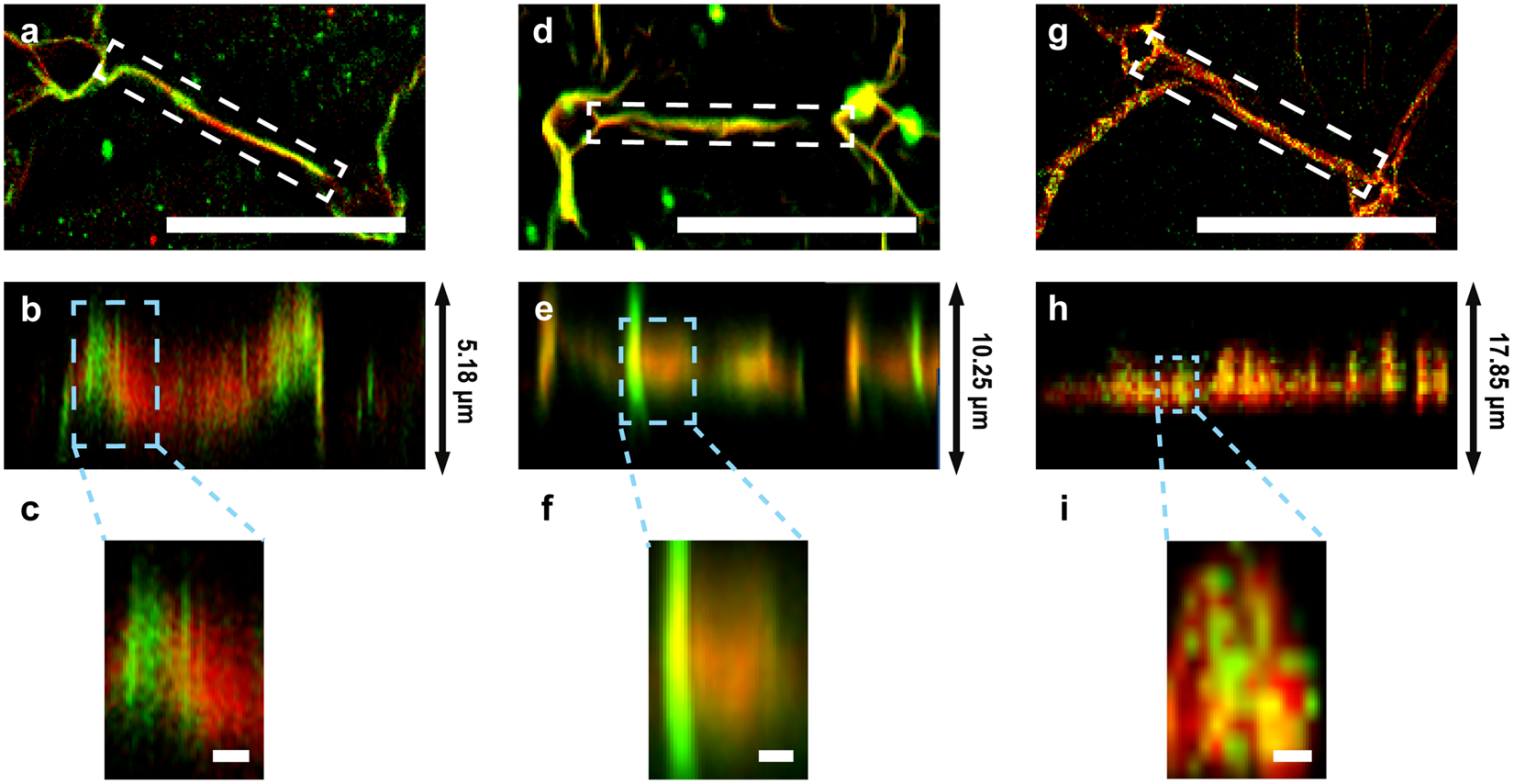
Confocal images of charge-induced self-assembly of composite FN and LAM fibers. (**a**–**f**) Images of composite FN/LAM fibers formed by sequential incubation on PSS micro-patterns: (**a**) Raw and (**b**) z-projected images of LAM (green) on PSS-induced FN fibers (red) and (**c**) magnified image from (**b**). (**d**) Raw and (**e**) z-projected images of FN (green) on PSS-induced LAM fibers (red) and (**f**) magnified image from (**e**). (**g**–**i**) Images of composite FN/LAM fibers formed by simultaneous incubation on 33% PSS micro-patterns: (**g**) Raw and (**h**) z-projected images of mixed FN (green) and LAM (red), and (**i**) magnified image from (**h**). The scales of (**a**,**d**, and **g**) are 50 µm, and those of (**c**,**f**, and **i**) are 1 µm.

When LAM or FN were sequentially incubated with the FN fibers (red, Fig. 2a–c and see Supplementary Video 1) or LAM fibers (red, Fig. 2d,f and see Supplementary Video 2), respectively, they were locally stacked on the pre-existing fibers. The magnified z-projected images (Fig. 2c,f) show that pre-incubation, proteins underwent self-induced fibrillogenesis to form fibrils, and that post-incubation, the new fibrils moved into empty spaces between the pre-existing protein’s fibrils, where they locally self-assembled to make continuous protein composite fibers, which we termed “locally stacked composite fibers.”

Surprisingly, simultaneous incubation of a mixture of FN and LAM formed composite FN/LAM fibers (Fig. 2g–i and see Supplementary Video 3). The homogeneous distribution of both proteins in these composite fibers (Fig. 2i) is not seen in locally stacked fibers and clearly indicates that FN and LAM fibrils were hetero-stacked during the initial spontaneous fibrillogenesis. We termed these hybridized fibers “hetero-stacked composite ECM fibers.” The hetero-stacking process results from chemical binding during the initial spontaneous fibrillogenesis, in contrast to the process that creates locally stacked fibers, which involves the insertion of non-hybridized self-assembled protein fibrils into pre-existing fibers.

The results demonstrated in Fig. 2 led us to inquire whether we could produce other types of composite ECM fibers by harnessing the protein-binding domains in ECM proteins, even when the proteins’ fibrillogenesis mechanisms differ. To address this question, we incubated COL and ELAS with pre-existing FN fibers because FN contains a COL-binding domain (I_6–9_)^34^ but not an ELAS-binding domain.

**Figure 3:**
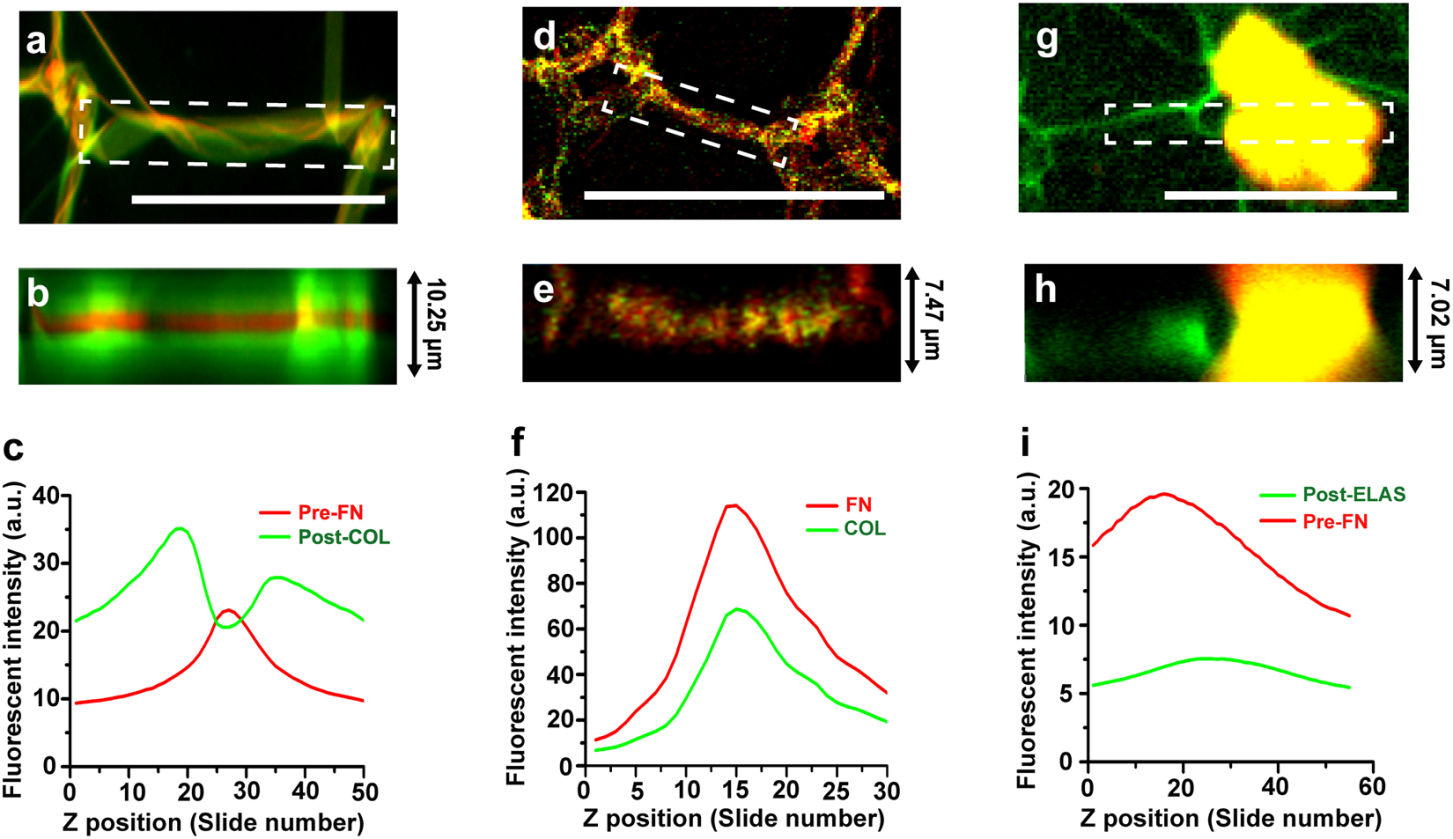
Confocal images of composite ECM fibers formed via binding sites. (**a**–**c**) Composite FN/COL fibers formed by sequential incubation on 33% PSS micro-patterns: (**a**) Raw and (**b**) z-projected confocal images of COL (green) on PSS-induced FN fibers (red), and (**c**) fluorescence intensity profile from (**b**) along the z direction. (**d**–**f**) Composite FN/COL fibers formed by simultaneous incubation on 33% PSS micro-patterns: (**d**) Raw and (**e**) z-projected confocal images of mixed COL (green) and FN (red) fibers, resulting in FN (red)-induced COL (green) fibers, and (**f**) fluorescence intensity profile from (**e**) along the z direction. (**g–i**) Sequential incubation of ELAS on FN fibers as a negative control: (**g**) Raw and (**h**) z-projected confocal images of ELAS (red) on pre-existing FN fibers (green), and (**i**) fluorescence intensity profile from (**h**) along the z direction. The scales of (**a**,**d**), and (**g**) are 50 µm.

When COL was sequentially incubated with pre-existing FN fibers, the FN fibers were coated with COL (Fig. 3a–c and see Supplementary Video 4) and formed a third type of composite ECM fiber, which we termed a “sandwiched composite fiber.” COL molecules accumulated on the surface of the FN fibers because they are sticky and strongly interact with suspended FN fibers.

In contrast, simultaneous incubation of FN and COL on the PSS patterns formed hetero-stacked composite fibers (Fig. 3d–f and see Supplementary Video 5) because COL cannot by itself form ordered networks on PSS micro-patterns (see Supplementary Fig. S1,i). Similar to the findings shown in Fig. 2g–i, both proteins were irregularly distributed throughout the fiber (Fig. 3i). As the FN fibers were induced on the PSS micro-patterns, FN molecules unfolded to expose COL binding sites, which led to the binding of COL to FN and initiated simultaneous self-induced assembly of the molecules into a single fiber.

As a negative control, ELAS was sequentially incubated on the FN substrate. The ELAS fibers were randomly adsorbed on the surface without interacting with the FN fibers (Fig. 3g–i, see Supplementary Fig. S1,j, and Videos) because ELAS fibrillogenesis relies neither on charge-initiated unfolding nor on FN-induced assembly. The fluorescence intensity profile in Fig. 3i does not reveal any interaction between FN and ELAS.

## Discussion

**Figure 4:**
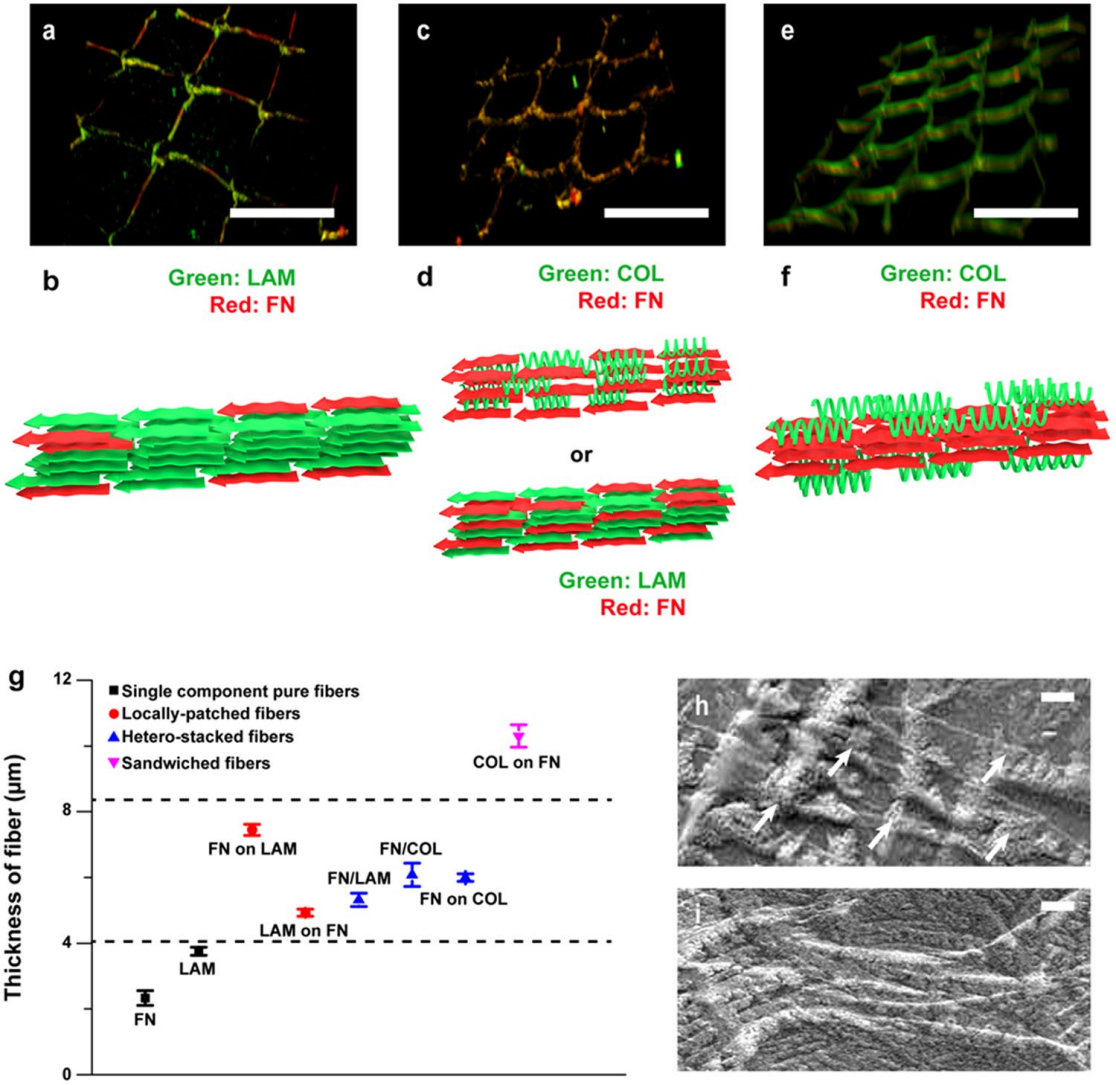
3D confocal images and SEM images with schematic animations. 3D-reconstructed z-stack images (upper panels) and molecular-level animations (middle panels) of (**a**,**b**) locally patched composite ECM fibers, (**c**,**d**) hetero-stacked composite ECM fibers, and (**e, f**) sandwiched composite ECM fibers of COL (green) and FN (red). The scales of (**a**,**c** and **e**) are 50 µm. (**g**) The graph shows the thickness of single-component and multi-component ECM protein fibers (n = 3; the error bars indicate the standard error of the mean). (**h**–**i**) SEM images of (**h**) LAM on FN and (**i**) COL on FN. White arrows indicate stacked single fibers or composite fibers. The scales of (**h**) and (**i**) are 1 µm.

Using both sequential and simultaneous incubations (Figs 2 and 3), we successfully engineered three types of composite ECM fibers: locally stacked composite fibers, hetero-stacked composite fibers, and sandwiched composite fibers (Fig. 4). Three-dimensionally (3D) reconstructed confocal images (Fig. 4a,b) show that sequential incubation of FN and LAM with pre-existing fibers formed locally patched fibers. Both the 3D-reconstructed z-stack images and the SEM images (Fig. 4h) of these structures show that binding domain-induced fibrils continuously assembled on the pre-existing fibers. Locally patched stacked were thicker than single-component ECM fibers due to the accumulation of protein fibrils (Fig. 4g).

In contrast, simultaneous incubation of FN and LAM on PSS micro-patterns produced hetero-stacked (i.e., β-sheet–β-sheet) composite ECM fibers because the β-sheet-rich FN and LAM underwent synchronized fibrillogenesis on the micro-patterns. The mechanism of the LAM/FN hetero-stacking during their simultaneous adsorption in a single fiber is obscure. It has been known, however, that the pre-existing LAM helps the self-assembly of FN^12^, then the charged-induced LAM or FN might mutually interact with FN or LAM molecules via the association of β-sheets in formation of the fibrils. Likewise, simultaneous incubation of FN and COL on PSS micro-patterns produced hetero-stacked (i.e., α-helix–β-sheet) composite ECM fibers. Because COL possese neither β-sheet in its secondary structure nor an unfolding mechanism, it cannot perform charge-initiated unfolding and self-assembly on PSS micro-patterns (see Supplementary Fig. S1,i). However, COL and FN can bind to each other and undergo synchronized fibrillogenesis because FN has a binding domain for COL^7^. While β-sheet rich FN and LAM molecules can mutually interact with each other, the helical COL molecules bound only to the exposed FN binding sites. Nevertheless, multiple FN domains in a single FN molecule allow the formation of insoluble fibers even with the structural interference of bound LAM or COL. The thickness of hetero-stacked fibers is similar to the thickness of locally stacked fibers owing to the homogeneous distribution of the constituent proteins in both cases (Fig. 4g).

Lastly, when COL was incubated with pre-existing FN fibers, COL covered the surface of the FN fibers owing to the specific FN-COL binding interaction (Fig. 4e,f). An SEM image (Fig. 4i) shows that COL fibers were adsorbed, not stacked, on the surface of the FN fibers. These fibers were significantly thicker than both single-component fibers and composite fibers because COL molecules accumulated on the outside surface of the FN fibers (Fig. 4g).

Deciphering the influence of different cell binding domains of the multi-composite ECM protein fibers on cell response is of great importance, in terms of biological and clicnical implications for the tissue specific ECM environment. We have confirmed that the PSS-induced FN fibers possessed excellent cell viability and resulted in functional tissue formation^28^. Subsequent and separate studies will further reveal how the binding sites and compositions of the multiple component fibers affect cell fate.

To the best of our knowledge, this is the first report of the production of multi-component ECM fibers via simultaneous or sequential incubations of ECM proteins chosen for their specific fibrillogenesis mechanisms and binding domains. Sequentially or simultaneouly incubating together any two of the FN, LAM, or COL proteins formed one of three different types of multi-component protein fibers: (1) locally stacked composite ECM fibers, (2) hetero-stacked composite ECM fibers, or (3) sandwiched composite ECM fibers. We found that ECM proteins could be used as fundamental units—like polymerizable monomers—to synthesize new multi-component fibers. We project that researchers will be able to use the fibrillogenesis mechanisms and binding domains in a variety of ECM proteins from diverse parts of the human body to create heterogeneous ECM architectures.

## Methods

### Preparation of ECM fibrillogenesis on spin-coated PSS surfaces and FT-IR analysis

Poly(styrene-co-4-styrene sulfonic acid) (33% PSS [mol% of SO_3_H: 33], Polymer Source, USA) was dissolved in dimethylformamide (Sigma Aldrich, Korea). For FTIR imaging, 33% PSS solutions were spin-coated onto gold-coated silicon wafers (Sigma) at 2000 rpm for 1 min. The PSS-coated surfaces were annealed at 150 °C overnight. After annealing, the surfaces were incubated with 66 µg/ml FN (Invitrogen, USA), LAM (Invitrogen, USA), type-Ι COL (Sigma Aldrich, Korea), or ELAS (Sigma Aldrich, Korea) in phosphate-buffered saline (pH 7.4) for 72 h at 37 °C. After incubation, the samples were washed with deionized water and dried in a desiccator. FT-IR images of the dried samples were acquired from the collected spectra, which were obtained using a Cary 660 spectrometer and a Cary 620 FT-IR microscope (Agilent Technologies, Korea). The resolution and total number of scans of the spectra were 4 cm^−1^ and 32, respectively, in the 800–4,000 cm^−1^ wavenumber regions with Agilent’s 16 × 16 focal plane array. Note that the background was corrected for each measurement.

### Preparation of single- and multi-component ECM fibers on PSS micro-patterns, sequential or simultaneous protein incubation, and confocal microscopy

PSS micro-patterns were prepared using the micro-contact printing technique described in our previous study^28^. Briefly, square arrays with star-like patterns were prepared by photolithography and soft lithography, and 33% PSS solution was inked onto polydimethylsiloxane (PDMS) stamps and then imprinted on coverslips. ECM proteins and polyvinyl acetate were sequentially or simultaneously incubated on the 33% PSS-patterned coverslips for 6 h and 72 h, respectively. After the final incubation, the samples were mounted on a microscope stage, and z-stacked images were obtained using a Zeiss LSM 5 LIVE confocal microscope.

### Image analysis

The z-stacked images were analyzed with Image J software (NIH). Z-projected images were obtained by the Image J volume viewer^35^. Three-dimensional animations of z-stacked images were compiled by the Image J 3D viewer^36,37^.

### Data availability

All data generated or analyzed during this study are included in this article (and its supplementary information files).

## Electronic supplementary material

Supplementary Video 1

Supplementary Video 2

Supplementary Video 3

Supplementary Video 4

Supplementary Video 5

Supplementary Video 6

Supplementary Information
