# Supplementary material for: Formation of Multi-Component Extracellular Matrix Protein Fibers
Source: Sci Rep. 2018 Jan 30;8:1913. doi: 10.1038/s41598-018-20371-8 (PMC5790006; doi:10.1038/s41598-018-20371-8)
Supplement: Supplementary file 7 — Supplementary Information [file 41598_2018_20371_MOESM7_ESM.pdf]

Supplementary Information

Formation of Multi-Component Extracellular Matrix Protein Fibers

*Seungkuk Ahn, Keel Yong Lee, Kevin Kit Parker, and Kwanwoo Shin**

Supplementary Figure

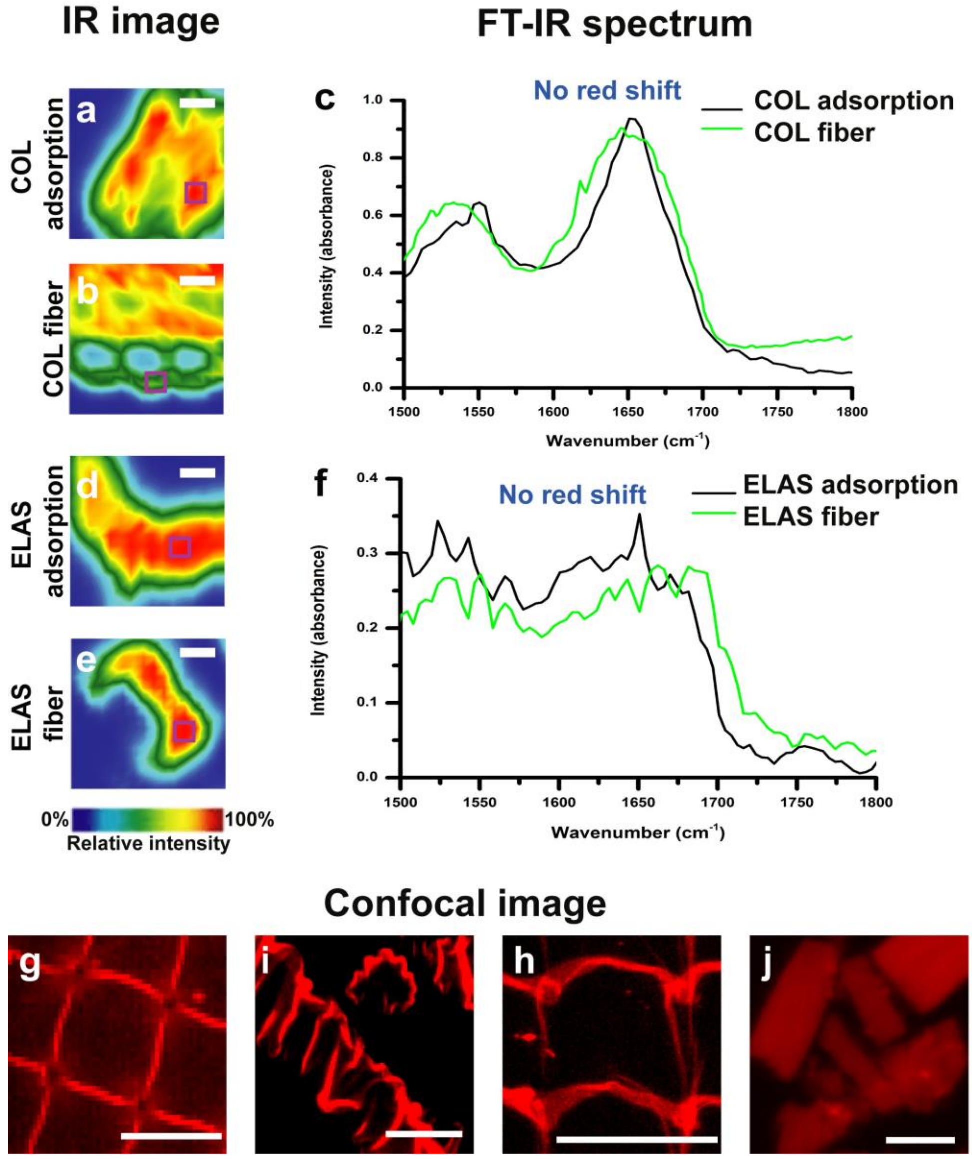

Figure S1: Single-component fibers incubated in an anionic environment. (a–f) Analysis of amide 1 peak shifts from IR images of adsorbed ECM proteins and ECM fibers formed by single incubation on a spin-coated 33% PSS surface: (a) COL adsorption, (b) COL fibers, and (c) FTIR spectra, where the scales of (a) and (b) are 20 µm. (d) ELAS adsorption, (e) adsorbed ELAS fibers, and (f) FTIR spectra, where the scales of (d) and (e) are 20 µm. (g–i) Confocal microscopy images of ECM protein fibers formed by single incubation on PSS micro-patterns: (g) FN and (h) LAM fibers, (i) anisotropic COL fibers, and (j) simply adsorbed ELAS fibers. The scales of (g), (h), (i),and (j) are 50, 50, 25, and 100 µm, respectively.

Supplementary Videos

Video 1. 3D view of LAM (green) on pre-existing FN fibers (red)

Video 2. 3D view of FN (green) on pre-existing LAM fibers (red)

Video 3. 3D view of a mixture of FN (green) and LAM (red) on PSS micro-patterns

Video 4. 3D view of COL (green)-coated FN (red) fibers

Video 5. 3D view of FN (red)-induced COL (green) fibers

Video 6. 3D view of ELAS (red) on pre-existing FN fibers (green)
